# Diacridinium hexa­chloridoplatinate(IV) dihydrate

**DOI:** 10.1107/S1600536810009566

**Published:** 2010-03-20

**Authors:** Kwang Ha

**Affiliations:** a School of Applied Chemical Engineering, The Research Institute of Catalysis, Chonnam National University, Gwangju 500-757, Republic of Korea

## Abstract

The asymmetric unit of the title compound, (C_13_H_10_N)_2_[PtCl_6_]·2H_2_O, contains a protonated acridine cation, one half of a [PtCl_6_]^2−^ dianionic complex and a solvent water mol­ecule. The octa­hedral [PtCl_6_]^2−^ dianion is located on an inversion centre. π–π inter­actions between neighboring acridinium cations produce stacks along the *a* axis; the shortest distance between the centroids of the six-membered rings within the cations is 3.553 (9) Å. In the crystal, two independent inter­molecular O—H⋯Cl hydrogen bonds, both involving the same Cl atom of the anion as acceptor, give rise to chains also running along the *a* axis; in addition each water mol­ecule, as a hydrogen-bond acceptor, is linked to the acridinium N—H group.

## Related literature

For related acridinium salts, see: Hafiz (2006 ▶); Veldhuizen *et al.* (1997 ▶). For the crystal structures of [PtCl_6_]^2−^ complexes, see: Karaca *et al.* (2009 ▶); Yousefi *et al.* (2007 ▶); Zordan & Brammer (2004 ▶).
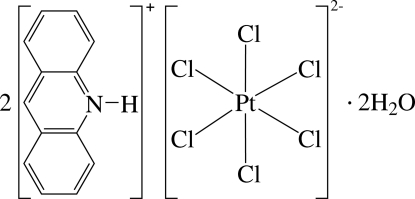


## Experimental

### 

#### Crystal data

(C_13_H_10_N)_2_[PtCl_6_]·2H_2_O
*M* *_r_* = 804.26
Triclinic, 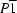

*a* = 7.4781 (8) Å
*b* = 9.8973 (10) Å
*c* = 10.7226 (12) Å
α = 70.675 (2)°
β = 71.505 (2)°
γ = 77.862 (2)°
*V* = 705.29 (13) Å^3^
*Z* = 1
Mo *K*α radiation
μ = 5.57 mm^−1^
*T* = 200 K
0.24 × 0.20 × 0.06 mm

#### Data collection

Bruker SMART 1000 CCD diffractometer
Absorption correction: multi-scan (*SADABS*; Bruker, 2000 ▶) *T*_min_ = 0.577, *T*_max_ = 1.000
4295 measured reflections
2725 independent reflections
2536 reflections with *I* > 2σ(*I*)
*R*_int_ = 0.029

#### Refinement

*R*[*F* ^2^ > 2σ(*F* ^2^)] = 0.059
*wR*(*F* ^2^) = 0.174
*S* = 1.21
2725 reflections
169 parameters
H-atom parameters constrained
Δρ_max_ = 1.99 e Å^−3^
Δρ_min_ = −2.96 e Å^−3^

### 

Data collection: *SMART* (Bruker, 2000 ▶); cell refinement: *SAINT* (Bruker, 2000 ▶); data reduction: *SAINT*; program(s) used to solve structure: *SHELXS97* (Sheldrick, 2008 ▶); program(s) used to refine structure: *SHELXL97* (Sheldrick, 2008 ▶); molecular graphics: *ORTEP-3* (Farrugia, 1997 ▶) and *PLATON* (Spek, 2009 ▶); software used to prepare material for publication: *SHELXL97*.

## Supplementary Material

Crystal structure: contains datablocks global, I. DOI: 10.1107/S1600536810009566/ya2119sup1.cif

Structure factors: contains datablocks I. DOI: 10.1107/S1600536810009566/ya2119Isup2.hkl

Additional supplementary materials:  crystallographic information; 3D view; checkCIF report

## Figures and Tables

**Table 1:** Hydrogen-bond geometry (Å, °)

*D*—H⋯*A*	*D*—H	H⋯*A*	*D*⋯*A*	*D*—H⋯*A*
N1—H1⋯O1^i^	0.88	1.94	2.782 (16)	161
O1—H1*A*⋯Cl1^ii^	0.84	2.74	3.485 (12)	149
O1—H1*B*⋯Cl1	0.84	2.62	3.342 (12)	145

Symmetry codes: (i) 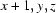; (ii) 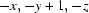.

**Fig. 1.**
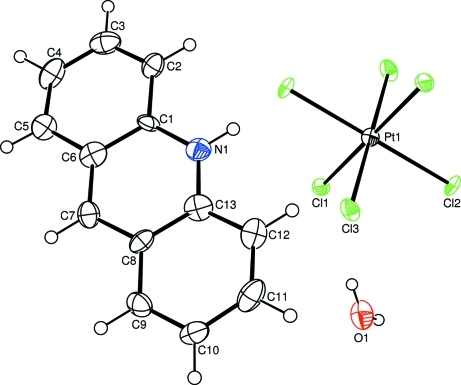
The structure of the title compound, with displacement ellipsoids drawn at the 50% probability level; H atoms are shown as small circles of arbitrary radius. Unlabelled atoms are related to the reference atoms by the (1-x, 1-y, -z) symmetry transformation.

**Fig. 2.**
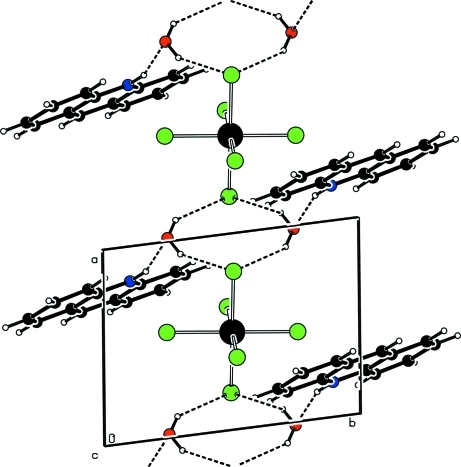
Packing diagram for the crystal of the title compound viewed down the c-axis; H-bonds are drawn as dashed lines.

## supplementary crystallographic information

### Comment

The asymmetric unit of the title compound, (C_13_H_10_N)_2_[PtCl_6_].2H_2_O, contains a protonated acridine cation, one half of a [PtCl_6_]^2-^ dianionic complex and a solvent water molecule (Fig. 1). The anion occupies a special position in the inversion centre; the Pt—Cl bond lengths, 2.370 (3), 2.377 (3) and 2.379 (3) Å, are similar to those found in other PtCl_6_ salts, i.e. (C_13_H_10_N)_2_[PtCl_6_].2C_2_H_6_OS (Karaca *et al.*, 2009), (C_14_H_13_N_2_)_2_[PtCl_6_] (Yousefi *et al.*, 2007) and (HPyX-3)_2_[PtCl_6_].2H_2_O (X = Br or I) (Zordan & Brammer, 2004).

The essentially planar acridinium cations [maximum deviation from the least-squares plane is equal to 0.025 (17) Å], are stacked in columns along the a-axis (Fig. 2); the shortest distance between the centroids of the six-membered rings in neighboring cations in the stack is equal to 3.553 (9) Å. Two independent O-H···Cl bonds, both involving atom Cl1 of the anion as acceptor (Table 1), give rise to the chains also running along the a-axis; in addition each water molecule, as an H-bond acceptor is linked to the acridinium N-H group (Fig. 2).

### Experimental

To a solution of K_2_PtCl_6_ (0.1999 g, 0.411 mmol) in H_2_O (20 ml) was added acridine (0.1548 g, 0.864 mmol) and the mixture was refluxed for 7 h. The precipitate was then separated by filtration, washed with water and acetone, and dried at 50 °C, to give an orange powder (0.2198 g). Crystals suitable for X-ray analysis were obtained by slow evaporation from a CH_3_CN solution.

### Refinement

H atoms were positioned geometrically and allowed to ride on their parent atoms [C—H = 0.95 Å, N—H = 0.88 Å and U_iso_(H) = 1.2U_eq_(C, N)]. The H atoms of the solvent water molecule were located from difference maps then allowed to ride on their parent O atom in the final cycles of refinement [O—H = 0.84 Å;  U_iso_(H)= 1.5U_eq_(O)]. The highest peak (1.99 e Å^-3^) and the deepest hole (-2.96 e Å^-3^) in the difference Fourier map are located 0.60 and 0.84 Å from the Cl3 and Pt1 atoms, respectively.

### Crystal data

**Table:**

(C_13_H_10_N)_2_[PtCl_6_]·2H_2_O	*Z* = 1
*M**_r_* = 804.26	*F*(000) = 390
Triclinic, *P*1	*D*_x_ = 1.894 Mg m^−^^3^
Hall symbol: -P 1	Mo *K*α radiation, λ = 0.71073 Å
*a* = 7.4781 (8) Å	Cell parameters from 2945 reflections
*b* = 9.8973 (10) Å	θ = 2.6–26.0°
*c* = 10.7226 (12) Å	µ = 5.57 mm^−^^1^
α = 70.675 (2)°	*T* = 200 K
β = 71.505 (2)°	Plate, orange
γ = 77.862 (2)°	0.24 × 0.20 × 0.06 mm
*V* = 705.29 (13) Å^3^	

### Data collection

**Table:**

Bruker SMART 1000 CCD diffractometer	2725 independent reflections
Radiation source: fine-focus sealed tube	2536 reflections with *I* > 2σ(*I*)
graphite	*R*_int_ = 0.029
φ and ω scans	θ_max_ = 26.0°, θ_min_ = 2.1°
Absorption correction: multi-scan (*SADABS*; Bruker, 2000)	*h* = −9→8
*T*_min_ = 0.577, *T*_max_ = 1.000	*k* = −12→12
4295 measured reflections	*l* = −13→9

### Refinement

**Table:**

Refinement on *F*^2^	Primary atom site location: structure-invariant direct methods
Least-squares matrix: full	Secondary atom site location: difference Fourier map
*R*[*F*^2^ > 2σ(*F*^2^)] = 0.059	Hydrogen site location: inferred from neighbouring sites
*wR*(*F*^2^) = 0.174	H-atom parameters constrained
*S* = 1.21	*w* = 1/[σ^2^(*F*_o_^2^) + (0.0248*P*)^2^ + 22.3646*P*] where *P* = (*F*_o_^2^ + 2*F*_c_^2^)/3
2725 reflections	(Δ/σ)_max_ < 0.001
169 parameters	Δρ_max_ = 1.99 e Å^−^^3^
0 restraints	Δρ_min_ = −2.96 e Å^−^^3^

### Special details

**Table:**

Geometry. All esds (except the esd in the dihedral angle between two l.s. planes) are estimated using the full covariance matrix. The cell esds are taken into account individually in the estimation of esds in distances, angles and torsion angles; correlations between esds in cell parameters are only used when they are defined by crystal symmetry. An approximate (isotropic) treatment of cell esds is used for estimating esds involving l.s. planes.
Refinement. Refinement of F^2^ against ALL reflections. The weighted R-factor wR and goodness of fit S are based on F^2^, conventional R-factors R are based on F, with F set to zero for negative F^2^. The threshold expression of F^2^ > 2sigma(F^2^) is used only for calculating R-factors(gt) etc. and is not relevant to the choice of reflections for refinement. R-factors based on F^2^ are statistically about twice as large as those based on F, and R- factors based on ALL data will be even larger.

### Fractional atomic coordinates and isotropic or equivalent isotropic displacement parameters (Å^2^)

**Table:**

	*x*	*y*	*z*	*U*_iso_*/*U*_eq_	
Pt1	0.5000	0.5000	0.0000	0.0289 (2)	
Cl1	0.1916 (4)	0.4938 (3)	0.1558 (3)	0.0282 (7)	
Cl2	0.3696 (4)	0.5171 (3)	−0.1801 (3)	0.0289 (7)	
Cl3	0.5401 (5)	0.2446 (3)	0.0506 (3)	0.0315 (7)	
N1	0.8227 (15)	0.1042 (13)	0.3453 (12)	0.035 (3)	
H1	0.8630	0.1656	0.2645	0.042*	
C1	0.8023 (17)	0.1451 (13)	0.4598 (13)	0.027 (3)	
C2	0.842 (2)	0.2841 (15)	0.4478 (15)	0.035 (3)	
H2	0.8838	0.3500	0.3600	0.043*	
C3	0.820 (2)	0.3208 (17)	0.5619 (18)	0.047 (4)	
H3	0.8465	0.4130	0.5553	0.056*	
C4	0.758 (2)	0.2231 (18)	0.6908 (16)	0.046 (4)	
H4	0.7445	0.2515	0.7699	0.055*	
C5	0.716 (2)	0.0926 (16)	0.7086 (16)	0.040 (3)	
H5	0.6727	0.0301	0.7982	0.048*	
C6	0.7368 (19)	0.0490 (15)	0.5907 (16)	0.037 (3)	
C7	0.694 (2)	−0.0880 (15)	0.6011 (14)	0.036 (3)	
H7	0.6495	−0.1540	0.6883	0.043*	
C8	0.7195 (18)	−0.1244 (15)	0.4794 (14)	0.032 (3)	
C9	0.678 (2)	−0.2605 (15)	0.4860 (15)	0.038 (3)	
H9	0.6376	−0.3289	0.5724	0.045*	
C10	0.698 (2)	−0.2930 (16)	0.3666 (16)	0.040 (3)	
H10	0.6665	−0.3824	0.3696	0.049*	
C11	0.765 (2)	−0.1910 (17)	0.2399 (17)	0.047 (4)	
H11	0.7788	−0.2149	0.1582	0.057*	
C12	0.810 (2)	−0.0609 (17)	0.2278 (15)	0.040 (3)	
H12	0.8583	0.0039	0.1405	0.048*	
C13	0.784 (2)	−0.0259 (14)	0.3493 (16)	0.037 (3)	
O1	0.0214 (17)	0.2503 (13)	0.0838 (11)	0.054 (3)	
H1A	−0.0687	0.3001	0.0522	0.081*	
H1B	0.1071	0.2824	0.0970	0.081*	

### Atomic displacement parameters (Å^2^)

**Table:**

	*U*^11^	*U*^22^	*U*^33^	*U*^12^	*U*^13^	*U*^23^
Pt1	0.0362 (4)	0.0291 (4)	0.0234 (4)	−0.0105 (3)	−0.0078 (3)	−0.0060 (3)
Cl1	0.0281 (16)	0.0351 (17)	0.0217 (14)	−0.0124 (13)	0.0031 (12)	−0.0124 (13)
Cl2	0.0365 (17)	0.0379 (17)	0.0221 (15)	−0.0061 (13)	−0.0158 (13)	−0.0125 (13)
Cl3	0.049 (2)	0.0154 (14)	0.0234 (15)	−0.0030 (13)	−0.0029 (13)	−0.0041 (12)
N1	0.024 (6)	0.043 (7)	0.039 (7)	−0.006 (5)	−0.008 (5)	−0.012 (5)
C1	0.023 (6)	0.027 (6)	0.028 (7)	−0.013 (5)	0.003 (5)	−0.007 (5)
C2	0.036 (8)	0.036 (8)	0.038 (8)	0.004 (6)	−0.018 (6)	−0.012 (6)
C3	0.042 (9)	0.035 (8)	0.072 (12)	−0.001 (7)	−0.024 (8)	−0.018 (8)
C4	0.049 (9)	0.055 (10)	0.046 (9)	0.014 (8)	−0.017 (7)	−0.036 (8)
C5	0.038 (8)	0.042 (8)	0.040 (8)	−0.001 (6)	−0.008 (6)	−0.016 (7)
C6	0.028 (7)	0.034 (7)	0.052 (9)	0.010 (6)	−0.017 (6)	−0.017 (7)
C7	0.036 (8)	0.035 (7)	0.032 (7)	0.003 (6)	−0.012 (6)	−0.006 (6)
C8	0.024 (7)	0.039 (8)	0.039 (8)	0.002 (5)	−0.015 (6)	−0.019 (6)
C9	0.040 (8)	0.027 (7)	0.042 (8)	−0.003 (6)	−0.016 (7)	0.000 (6)
C10	0.043 (8)	0.037 (8)	0.051 (9)	−0.006 (6)	−0.019 (7)	−0.017 (7)
C11	0.056 (10)	0.048 (9)	0.051 (10)	0.015 (8)	−0.027 (8)	−0.031 (8)
C12	0.032 (8)	0.047 (9)	0.039 (8)	0.010 (6)	−0.007 (6)	−0.020 (7)
C13	0.034 (8)	0.024 (7)	0.053 (9)	0.012 (6)	−0.018 (7)	−0.012 (6)
O1	0.052 (7)	0.064 (8)	0.039 (6)	−0.005 (6)	−0.008 (5)	−0.012 (6)

### Geometric parameters (Å, °)

**Table:**

Pt1—Cl2^i^	2.370 (3)	C5—C6	1.42 (2)
Pt1—Cl2	2.370 (3)	C5—H5	0.9500
Pt1—Cl3^i^	2.377 (3)	C6—C7	1.42 (2)
Pt1—Cl3	2.377 (3)	C7—C8	1.412 (19)
Pt1—Cl1^i^	2.379 (3)	C7—H7	0.9500
Pt1—Cl1	2.379 (3)	C8—C13	1.42 (2)
N1—C13	1.362 (18)	C8—C9	1.418 (19)
N1—C1	1.370 (17)	C9—C10	1.38 (2)
N1—H1	0.8800	C9—H9	0.9500
C1—C6	1.411 (19)	C10—C11	1.41 (2)
C1—C2	1.424 (18)	C10—H10	0.9500
C2—C3	1.34 (2)	C11—C12	1.36 (2)
C2—H2	0.9500	C11—H11	0.9500
C3—C4	1.40 (2)	C12—C13	1.40 (2)
C3—H3	0.9500	C12—H12	0.9500
C4—C5	1.33 (2)	O1—H1A	0.8400
C4—H4	0.9500	O1—H1B	0.8400
			
Cl2^i^—Pt1—Cl2	180.0	C3—C4—H4	118.2
Cl2^i^—Pt1—Cl3^i^	89.44 (11)	C4—C5—C6	118.4 (15)
Cl2—Pt1—Cl3^i^	90.56 (11)	C4—C5—H5	120.8
Cl2^i^—Pt1—Cl3	90.56 (11)	C6—C5—H5	120.8
Cl2—Pt1—Cl3	89.44 (11)	C1—C6—C7	119.4 (13)
Cl3^i^—Pt1—Cl3	180.00 (16)	C1—C6—C5	118.8 (13)
Cl2^i^—Pt1—Cl1^i^	90.27 (11)	C7—C6—C5	121.8 (14)
Cl2—Pt1—Cl1^i^	89.73 (11)	C8—C7—C6	118.7 (13)
Cl3^i^—Pt1—Cl1^i^	90.55 (11)	C8—C7—H7	120.6
Cl3—Pt1—Cl1^i^	89.45 (11)	C6—C7—H7	120.6
Cl2^i^—Pt1—Cl1	89.73 (11)	C7—C8—C13	120.8 (13)
Cl2—Pt1—Cl1	90.27 (11)	C7—C8—C9	120.3 (13)
Cl3^i^—Pt1—Cl1	89.45 (11)	C13—C8—C9	118.9 (12)
Cl3—Pt1—Cl1	90.55 (11)	C10—C9—C8	119.7 (13)
Cl1^i^—Pt1—Cl1	180.00 (10)	C10—C9—H9	120.1
C13—N1—C1	123.9 (12)	C8—C9—H9	120.1
C13—N1—H1	118.0	C9—C10—C11	118.7 (13)
C1—N1—H1	118.0	C9—C10—H10	120.6
N1—C1—C6	119.2 (11)	C11—C10—H10	120.6
N1—C1—C2	120.8 (12)	C12—C11—C10	123.9 (14)
C6—C1—C2	119.9 (12)	C12—C11—H11	118.1
C3—C2—C1	119.3 (14)	C10—C11—H11	118.1
C3—C2—H2	120.4	C11—C12—C13	117.3 (15)
C1—C2—H2	120.4	C11—C12—H12	121.4
C2—C3—C4	119.9 (14)	C13—C12—H12	121.4
C2—C3—H3	120.0	N1—C13—C12	120.7 (14)
C4—C3—H3	120.0	N1—C13—C8	117.9 (13)
C5—C4—C3	123.7 (14)	C12—C13—C8	121.4 (13)
C5—C4—H4	118.2	H1A—O1—H1B	125.9
			
C13—N1—C1—C6	−0.4 (19)	C6—C7—C8—C13	−1(2)
C13—N1—C1—C2	−178.9 (12)	C6—C7—C8—C9	180.0 (12)
N1—C1—C2—C3	179.7 (13)	C7—C8—C9—C10	177.9 (13)
C6—C1—C2—C3	1(2)	C13—C8—C9—C10	−1(2)
C1—C2—C3—C4	0(2)	C8—C9—C10—C11	2(2)
C2—C3—C4—C5	−1(2)	C9—C10—C11—C12	−1(2)
C3—C4—C5—C6	1(2)	C10—C11—C12—C13	−2(2)
N1—C1—C6—C7	0.3 (19)	C1—N1—C13—C12	−179.3 (12)
C2—C1—C6—C7	178.8 (12)	C1—N1—C13—C8	−0.1 (19)
N1—C1—C6—C5	−179.8 (12)	C11—C12—C13—N1	−178.5 (13)
C2—C1—C6—C5	−1.3 (19)	C11—C12—C13—C8	2(2)
C4—C5—C6—C1	0(2)	C7—C8—C13—N1	0.6 (19)
C4—C5—C6—C7	−179.7 (14)	C9—C8—C13—N1	179.9 (12)
C1—C6—C7—C8	0.2 (19)	C7—C8—C13—C12	179.8 (13)
C5—C6—C7—C8	−179.7 (13)	C9—C8—C13—C12	−1(2)

Symmetry codes: (i) −*x*+1, −*y*+1, −*z*.

### Hydrogen-bond geometry (Å, °)

**Table:**

*D*—H···*A*	*D*—H	H···*A*	*D*···*A*	*D*—H···*A*
N1—H1···O1^ii^	0.88	1.94	2.782 (16)	161
O1—H1A···Cl1^iii^	0.84	2.74	3.485 (12)	149
O1—H1B···Cl1	0.84	2.62	3.342 (12)	145

Symmetry codes: (ii) *x*+1, *y*, *z*; (iii) −*x*, −*y*+1, −*z*.
